# Supplementary material for: Pore partition in two-dimensional covalent organic frameworks
Source: Nat Commun. 2023 Jun 8;14:3360. doi: 10.1038/s41467-023-39126-9 (PMC10250421; doi:10.1038/s41467-023-39126-9)
Supplement: Supplementary file 1 — Supplementary Information [file 41467_2023_39126_MOESM1_ESM.pdf]

Supplementary Information

Pore partition in two-dimensional covalent organic frameworks

Xiaoyi Xu[1], Xinyu Wu[1], Kai Xu[1], Hong Xu[2], Hongzheng Chen[1] & Ning Huang[1*]

[1]*State Key Laboratory of Silicon and Advanced Semiconductor Materials, International Research Center for X Polymers, Department of Polymer Science and Engineering, Zhejiang University, Hangzhou 310027, China.*

[2]*Institute of Nuclear and New Energy Technology, Tsinghua University, Beijing 100084, China.*

*Corresponding Author: Prof. Dr. Ning Huang (nhuang@zju.edu.cn)

Table of Contents

Materials and Methods--S3

Supplementary Figures--S8

Supplementary Tables--S42

Supplementary References---S48

Materials and Methods

^1H NMR spectra were measured on Bruker AVANCE III 400 NMR spectrometer, where chemical shifts (δ in ppm) were determined with a residual proton of the solvent as standard. Matrix-assisted laser desorption ionization time-of-flight mass (MALDI-TOF MS) spectra were recorded on an Applied Biosystems BioSpectrometry model Voyager-DE-STR spectrometer in reflector or linear mode. ICP-MS was performed on a Perkin-Elmer Elan DRC II quadrupole inductively coupled plasma mass spectrometer analyzer. Fourier transform infrared (FT-IR) spectra were recorded on a Nicolet 6700 infrared spectrometer. X-ray diffraction (XRD) data were recorded on a PANalytical X'Pert PRO diffractometer by depositing powder on glass substrate, from $2\theta = 1.5°$ up to 40° with 0.02° increment. Elemental analysis was performed on a Euro Vector EA3000 elemental analyzer. TGA measurements were performed on a Mettler-Toledo model TGA/SDTA851e under N_2, by heating to 800 °C at a rate of 10 °C min^{-1}.

Scanning electron microscope (SEM) images were collected using a Hitachi S-4800 system. Transmission electron microscope (TEM) images were obtained with a JEM-2100F, JEOL system. Solid state nuclear magnetic resonance ^{13}C cross polarization magic angle spinning nuclear magnetic resonance spectra (^{13}C CP MAS NMR) were recorded on a JEOL JNM ECA600 MHz, 3.2 mm rotor, MAS of 20 kHz, recycle delay of 1 sec. Nitrogen sorption isotherms were measured at 77 K with a Micromeritics Instrument Corporation model 3Flex surface characterization analyzer. Before measurement, the samples were degassed in vacuum at 100 °C for more than 10 h. Brunauer-Emmett-Teller (BET) method was utilized to calculate the specific surface areas. By using the non-local density functional theory (NLDFT) model, the pore volume was derived from the sorption curve.

All the chemicals commercially available were used without further purification. 4-ethynylbenzene (>98%), 4-iodoaniline (>97%), octanoyl chloride (>99%), $Pd(PPh_3)_4$ (>9.2%Pd), CuI (>99%), $Co_2(CO)_8$ (>95%, stabilized with 1-5% hexane), veratrole (>98%), terephthalaldehyde (>98%), 1,3,5-benzenetriboronic acid (>98%) and HBr (48 wt% in water), were purchased from Adamas. Pyridine (AR), dichloromethane (AR), triethylamine (AR), tetrahydrofuran (AR), acetone (AR), methanol (AR), 1,4-dioxane (AR), mesitylene (AR), N,N-dimethylformamide (AR), ethyl ether (AR), HCl (AR), H_2SO_4 (AR), NaOH (AR), and ammonia (AR) were purchased from Sinopharm Chemical Reagent Co., Ltd.

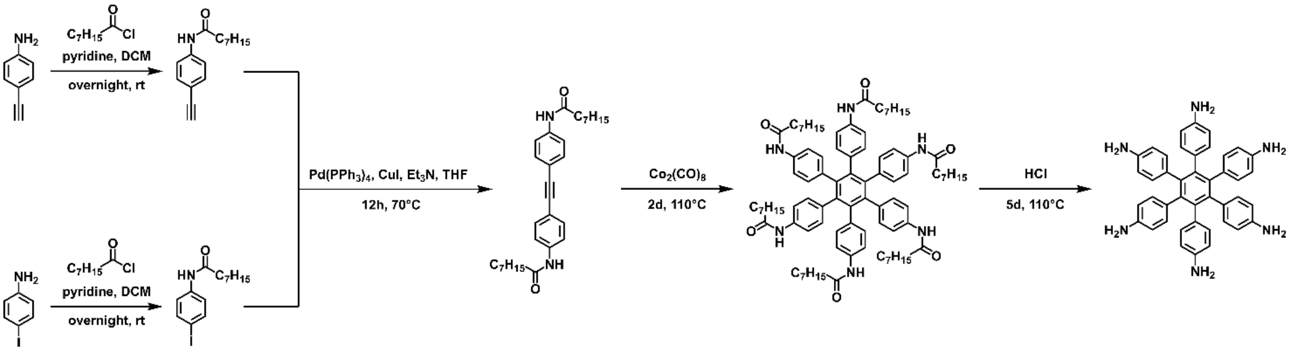

Synthesis of octanoylamino-4-ethynylbenzene. In a 100 mL round bottom flask equipped with a magnetic stir bar, a mixture of 4-ethynylbenzene (1.97 g, 16.86 mmol) and octanoyl chloride (3.02 g, 18.55 mmol) was dissolved in dichloromethane (60 mL). Afterwards, pyridine (2 g, 25.28 mmol) was added, and the reaction mixture was stirred overnight at room temperature. The reaction progress was monitored by TLC. After full conversion, the mixture was extracted with 1M HCl aqueous solution, 1M NaOH aqueous solution and brine. The organic solution was removed under reduced pressure. After evaporation, the resulting product was dried at 40 °C for 18 h to give octanoylamino-4-ethynylbenzene as a beige solid (3.77 g, 92%) and pure enough for the next step. ^{1}H NMR (400 MHz, Chloroform-*d*) δ 7.50 (d, J = 8.5 Hz, 2H), 7.44 (d, J = 8.1 Hz, 2H), 7.17 (s, 1H), 3.04 (d, J = 2.9 Hz, 1H), 2.36 (t, J = 7.7 Hz, 2H), 1.72 (t, J = 7.5 Hz, 2H), 1.34 – 1.21 (m, 8H), 0.87 (t, J = 6.7 Hz, J = 7.0 Hz, 3H). ^{13}C NMR (101 MHz, Chloroform-*d*) δ 171.48, 138.47, 132.92, 127.13, 117.57, 83.39, 83.38, 37.85, 31.65, 29.20, 29.00, 25.53, 22.58, 14.01. HRMS (m/z): $[M+H]^+$ calcd. for $C_{16}H_{21}NO$ 243.1600; found 243.3560.

Synthesis of octanoylamino-4-iodobenzene. In a 100 mL round bottom flask equipped with a magnetic stir bar, a mixture of 4-iodoaniline (3.69 g, 16.86 mmol) and octanoyl chloride (3.02 g, 18.55 mmol) was dissolved in dichloromethane (60 mL). Afterwards, pyridine (2 g, 25.28 mmol) was added, and the reaction mixture was stirred overnight at room temperature. The reaction progress was monitored by TLC. After full conversion, the mixture was extracted with 1M HCl aqueous solution, 1M NaOH aqueous solution and brine. The organic solution was removed under reduced pressure. After evaporation, the resulting product was dried at 40 °C for 18 h to give octanoylamino-4-iodobenzene as a pale-yellow solid (5.59 g, 96%) and pure enough for the next step. ^{1}H NMR (400 MHz, Chloroform-*d*) δ 7.61 (d, J = 8.5 Hz, 2H), 7.31 (d, J = 8.4 Hz, 2H), 7.14 (s, 1H), 2.34 (t, J =

7.5 Hz, 2H), 1.75 – 1.68 (m, 2H), 1.31 (d, J = 17.3 Hz, 8H), 0.88 (t, J = 6.7 Hz, J = 7.0 Hz, 3H). ^{13}C NMR (101 MHz, Chloroform-d) δ 171.66, 137.96, 137.82, 121.71, 87.08, 37.76, 31.65, 29.21, 29.01, 25.53, 22.58, 14.03. HRMS (m/z): $[M+H]^+$ calcd. for $C_{16}H_{20}INO$ 345.0600; found 345.2200.

Synthesis of bis[4-octanoylamino-phenyl] acetylene. In a 100 mL round bottom flask equipped with a magnetic stir bar, a mixture of octanoylamino-4-iodobenzene (3.45 g, 10.00 mmol), octanoylamino-4-ethynylbenzene (2.43 g, 10.00 mmol), $Pd(PPh_3)_4$ (577.80 mg, 0.50 mmol), and CuI (9.49 mg, 0.50 mmol) were dissolved in dry tetrahydrofuran (40 mL). Afterwards, triethylamine (10 mL) was added and the reaction mixture was stirred for 12 h at 70 °C. The solution was reduced to a small amount under reduced pressure. After filtration, the resulting precipitate was washed with methanol to give bis[4-octanoylamino-phenyl] acetylene as a silver solid (3.59 g, 78%) and pure enough for the next step.^{1}H NMR (400 MHz, DMSO-d_6) δ 10.06 (s, 2H), 7.65 (d, J = 8.6 Hz, 4H), 7.45 (d, J = 8.6 Hz, 4H), 2.32 (t, J = 7.3 Hz, J = 7.6 Hz, 4H), 1.59 (t, J = 7.3 Hz, J = 7.0 Hz, 4H), 1.28 (m, 16H), 0.87 (t, J = 6.7 Hz, J = 7.0 Hz s, 6H). ^{13}C NMR (101 MHz, DMSO-d_6) δ 172.07, 140.06, 132.38, 119.41, 117.12, 89.17, 36.99, 31.69, 29.14, 28.99, 25.58, 22.59, 14.48. HRMS (m/z): $[M+H]^+$ calcd. for $C_{30}H_{40}N_2O_2$ 460.3100; found 460.6610.

Synthesis of hexa(octanoylamino phenyl) benzene. In a 500 mL round bottom flask equipped with a magnetic stir bar, bis[4-octanoylamino-phenyl] acetylene (6.69 g, 14.52 mmol) and $Co_2(CO)_8$ (496.59 mg, 1.45 mmol) was mixed with dry 1,4-dioxane (230 mL). Afterwards, the mixture was heated at 110 °C for 48 h under N_2. After cooling to room temperature, ethyl ether was added to the reaction mixture. After filtration, the residue was dissolved in N,N-dimethylformamide. After filtration, the filtrate was added to H_2O. After filtration, the resulting precipitate was washed with H_2O and methanol to give hexa (octanoylamino phenyl) benzene as a white solid (3.72 g, 55.6 %) and pure enough for the next step.^{1}H NMR (400 MHz, DMSO-d_6) δ 9.55 (s, 6H), 7.10 (d, J = 8.5 Hz, 12H), 6.73 (d, J = 8.3 Hz, 12H), 2.17 (s, 12H), 1.50 (d, J = 7.6 Hz, 12H), 1.23 (s, 48H), 0.84 (t, J = 6.4 Hz, 18H). ^{13}C NMR (101 MHz, DMSO-d_6) δ 170.88, 139.90, 136.34, 135.01, 131.02, 116.91, 36.37, 31.07, 28.66, 28.38, 24.87, 21.98, 13.84. HRMS (m/z): $[M+H]^+$ calcd. for $C_{90}H_{120}N_6O_6$ 1380.9300; found 1381.9900.

Synthesis of hexaaminophenyl benzene. In a 500 mL round bottom flask equipped with a magnetic stir bar, hexa (octanoylamino phenyl) benzene (5 g, 3.62 mmol) was dispersed in hydrochloric acid

aqueous solution (300 mL). Afterwards, the mixture was heated at 100 °C for 5d under N_2. After cooling to room temperature, 1M NaOH aqueous solution was added to the reaction mixture. After filtration, the resulting precipitate was washed with ethyl ether and methanol to give hexaaminophenyl benzene as an off-white solid (1.51 g, 67%) and pure enough for the COF synthesis. ^{1}H NMR (400 MHz, DMSO-d_6) δ 6.36 (d, J = 8.0 Hz, 12H), 6.04 (d, J = 8.0 Hz, 12H), 4.52 (s, 12H). ^{13}C NMR (101 MHz, DMSO-d_6) δ 144.48, 140.23, 131.46, 129.83, 112.64. HRMS (m/z): $[M+H]^+$ calcd. for $C_{42}H_{36}N_6$ 624.3000; found 624.7900.

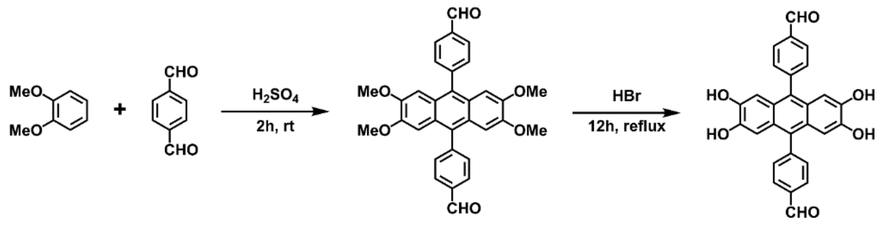

Synthesis of 4,4'-(2,3,6,7-tetramethoxy-9,10-anthracenediyl)bis[benzaldehyde]. In a 50 ml round-bottom flask equipped with a magnetic stir bar, a solution of veratrole (1.38 g, 10.00 mmol) and terephthalaldehyde (2.68 g, 20.00 mmol) in dichloromethane (5 mL) was added dropwise to 84% sulfuric acid (10 mL) at 0 °C. After addition, the suspension was stirred for 2h at room temperature. The reaction progress was monitor by TLC. After full conversion was achieved, the reaction was quenched with water and neutralized by ammonia. After extraction with dichloromethane, the solvent was removed under reduced pressure. The crude product by column chromatography on silica gel, elution with petroleum ether/ dichloromethane. After evaporation, the resulting product was dried at 80 °C for 12 h to give 4,4'-(2,3,6,7-tetramethoxy-9,10-anthracenediyl) bis[benzaldehyde] as a light-yellow solid (1.57 g, 62%) and pure enough for the next step. ^{1}H NMR (400 MHz, $CDCl_3$) δ 10.21 (s, 2H), 8.16 (d, J = 8.1 Hz, 4H), 7.69 (d, J = 8.0 Hz, 4H), 6.70 (s, 4H), 3.72 (s, 12H). ^{13}C NMR (101 MHz, $CDCl_3$) δ 192.15, 149.31, 146.70, 135.67, 132.01, 131.91, 130.23, 125.36, 103.37, 55.58. HRMS (m/z): $[M+H]^+$ calcd. for $C_{32}H_{26}O_6$ 506.1700; found 506.5500.

Synthesis of 4,4'-(2,3,6,7-tetrahydroxide -9,10-anthracenediyl)bis[benzaldehyde]. In a 100 ml round-bottom flask equipped with a magnetic stir bar, a solution of 4,4'-(2,3,6,7-tetramethoxy-9,10-anthracenediyl) bis[benzaldehyde] (1.00 g, 1.97 mmol) was dispersed in hydrobromic acid aqueous solution (10 mL). The reaction mixture was stirred at reflux overnight and a large amount of green

solid formed. After filtration, the resulting product was dried at 40 °C for 12 h to give 4,4'-(2,3,6,7-tetrahydroxide-9,10-anthracenediyl) bis[benzaldehyde] as a yellow green solid (0.59 g, 69%) and pure enough for the COF synthesis. ^{1}H NMR (400 MHz, DMSO-d_6) δ 10.21 (s, 2H), 9.47 (s, 4H), 8.18 (d, J = 8.1 Hz, 4H), 7.64 (d, J = 8.1 Hz, 4H), 6.62 (s, 4H). ^{13}C NMR (101 MHz, DMSO-d_6) δ 193.05, 146.78, 146.57, 135.19, 131.90, 129.88, 129.52, 124.57, 106.06. HRMS (m/z): $[M+H]^+$ calcd. for $C_{28}H_{18}O_6$ 450.1100; found 450.1920.

Supplementary Figures

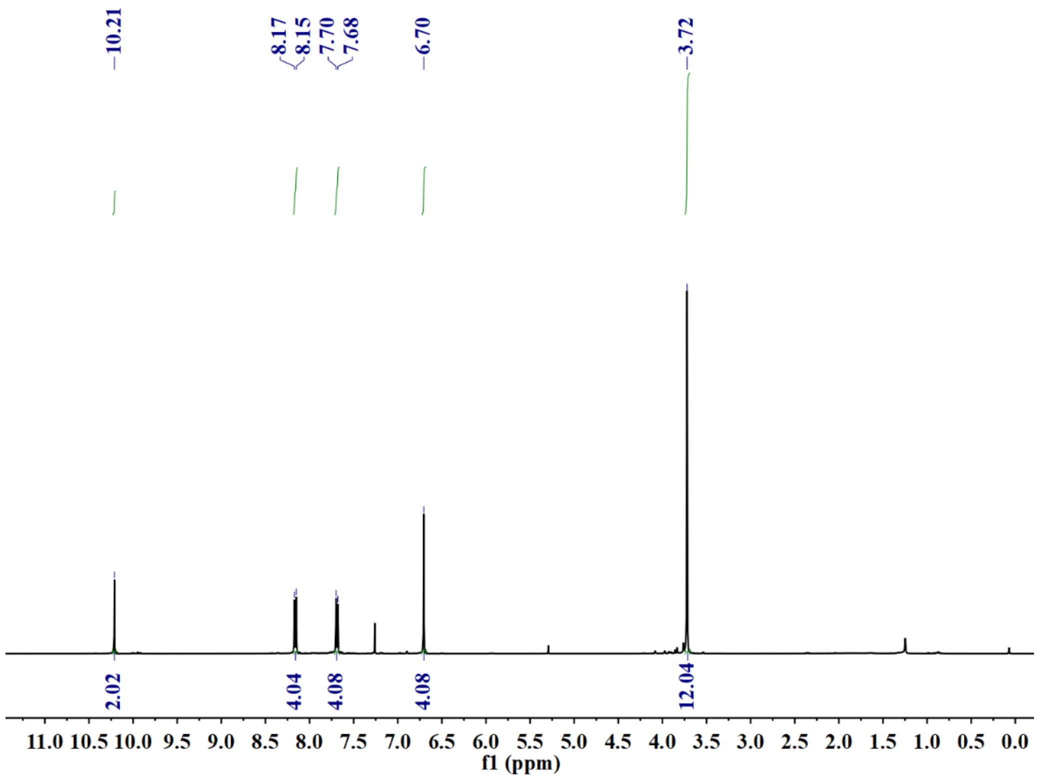

Supplementary Fig. 1 | NMR spectra. ^{1}H NMR spectrum of 4,4'-(2,3,6,7-tetramethoxy-9,10-anthracenediyl)bis[benzaldehyde].

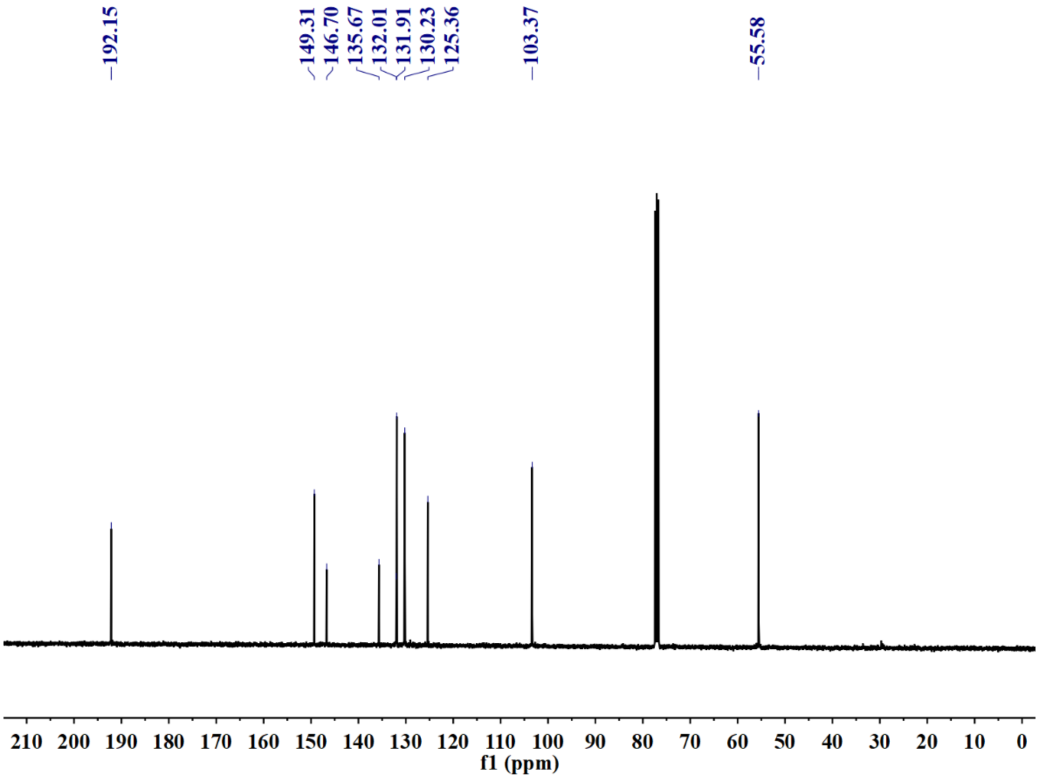

Supplementary Fig. 2 | NMR spectra. ^{13}C NMR spectrum of 4,4'-(2,3,6,7-tetramethoxy-9,10-anthracenediyl)bis[benzaldehyde].

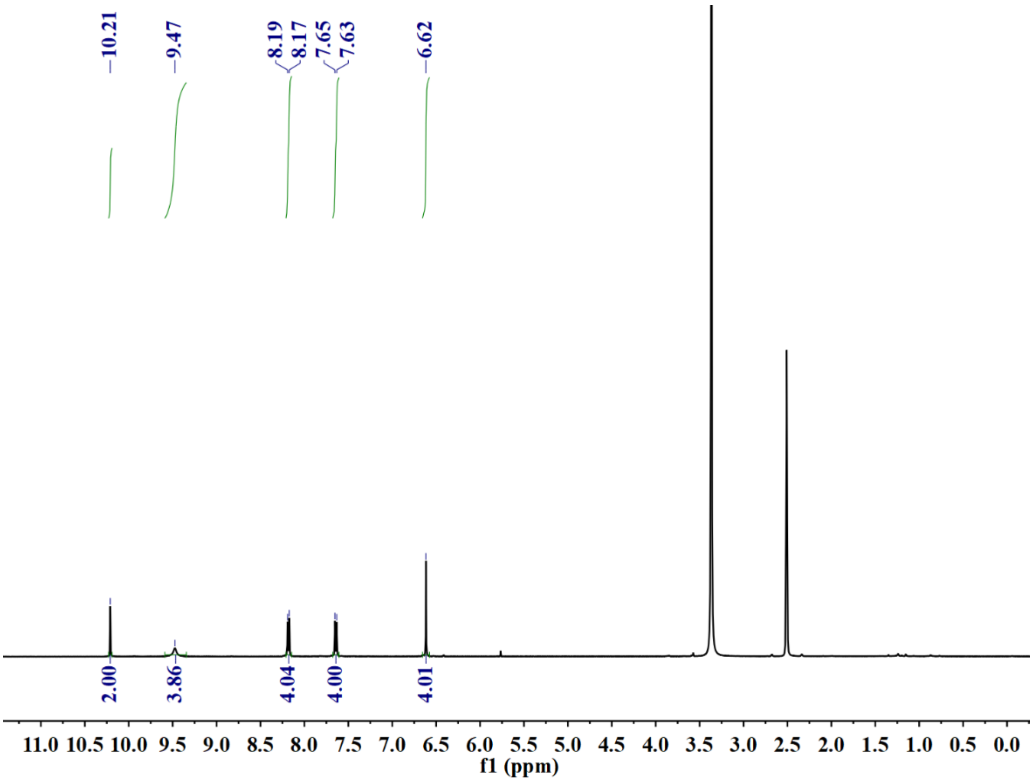

Supplementary Fig. 3 | NMR spectra. ^{1}H NMR spectrum of 4,4'-(2,3,6,7-tetramethoxy-9,10-anthracenediyl)bis[benzaldehyde].

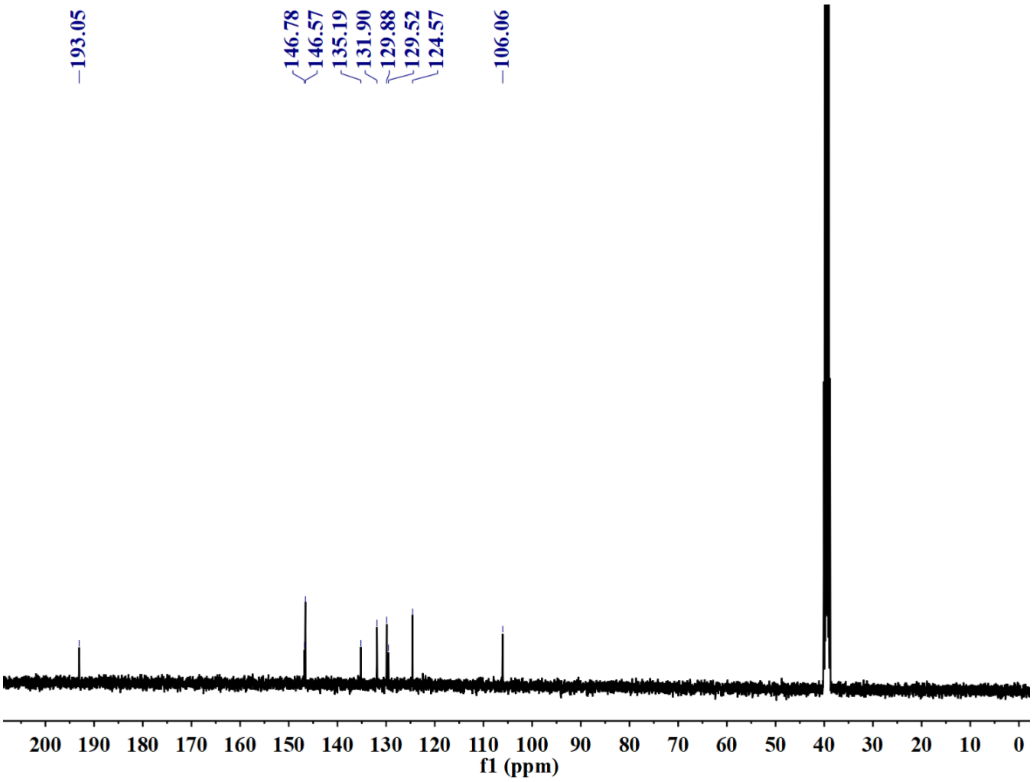

Supplementary Fig. 4 | NMR spectra. ^{13}C NMR spectrum of 4,4'-(2,3,6,7-tetramethoxy-9,10-anthracenediyl)bis[benzaldehyde].

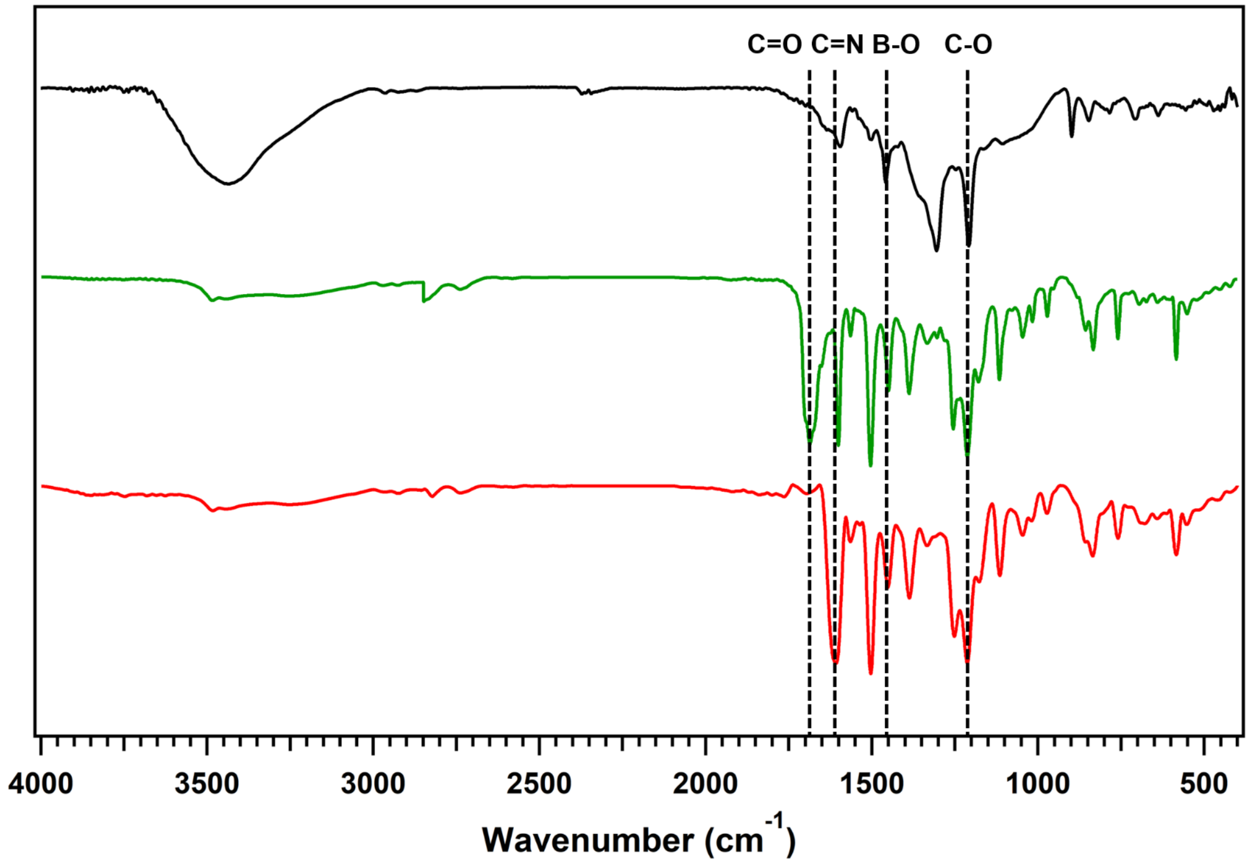

Supplementary Fig. 5 | IR spectra. IR spectra of Ph-An-COF (black curve), DBAAn-BTBA-COF (green curve), and DBAAn-BTBA-HAPB-COF (red curve). The adsorption peaks at 1,387 and 1,334 cm^{-1} correspond to the vibration bands of boronate rings. The band at 1,242 cm^{-1} represents the C–O vibration band. The strong vibration band at 1,622 cm^{-1} was attributed to the C=N bond. The C=O vibration band at 1,698 cm^{-1} in DBAAn-BTBA-HAPB-COF disappeared, elucidating all the aldehyde groups were transformed into C=N bonds.

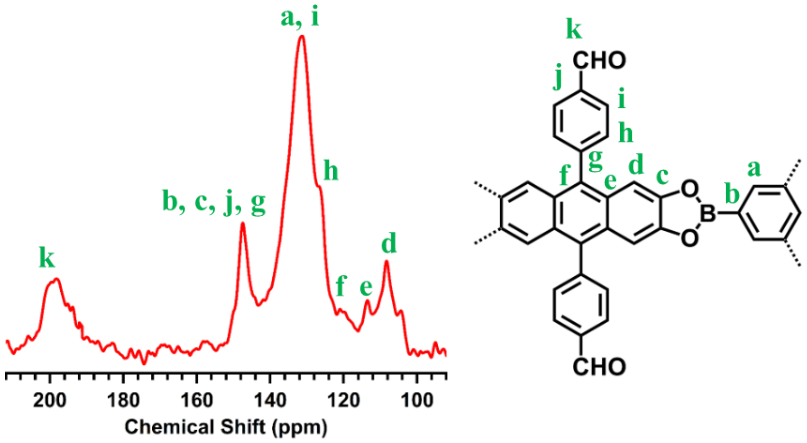

Supplementary Fig. 6 | NMR spectrum. Solid-state ^{13}C CP/MAS nuclear magnetic resonance spectroscopy of DBAAn-BTBA-COF. The resonances at 108.3, 114.7, 123.6, 126.8, 131.5, 147.5, 198.6 ppm are characteristic signals for carbon atoms in DBAAn-BTBA-COF.

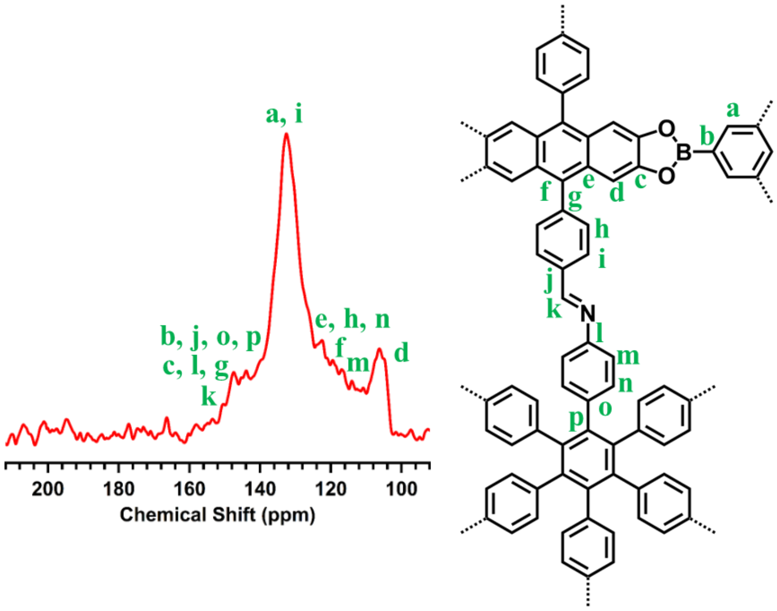

Supplementary Fig. 7 | NMR spectrum. Solid-state ^{13}C CP/MAS nuclear magnetic resonance spectroscopy of DBAAn-BTBA-HAPB-COF. The resonances at 106.7, 114.1, 115.8, 119.4, 122.5, 132.5, 143.9, 147.5, 149.2, 151.3, and 153.4 ppm are characteristic signals for carbon atoms in DBAAn-BTBA-HAPB-COF.

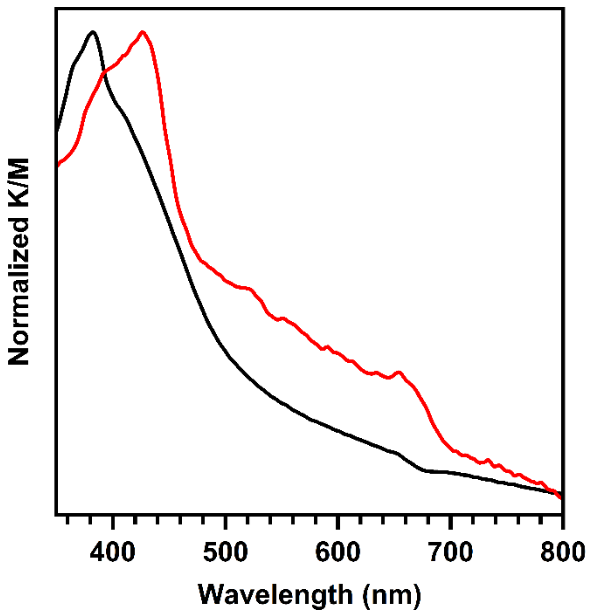

Supplementary Fig. 8 | UV-vis spectra. UV-vis spectra of DBAAn-BTBA-COF (black curve) and DBAAn-BTBA-HAPB-COF (red curve).

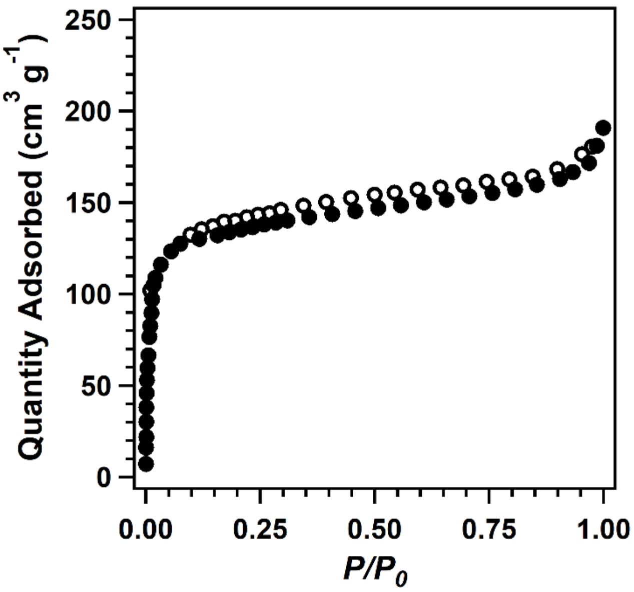

Supplementary Fig. 9 | Porosity. Nitrogen sorption isotherm of DBAAn-BTBA-HAPB-COF synthesized using one-pot method.

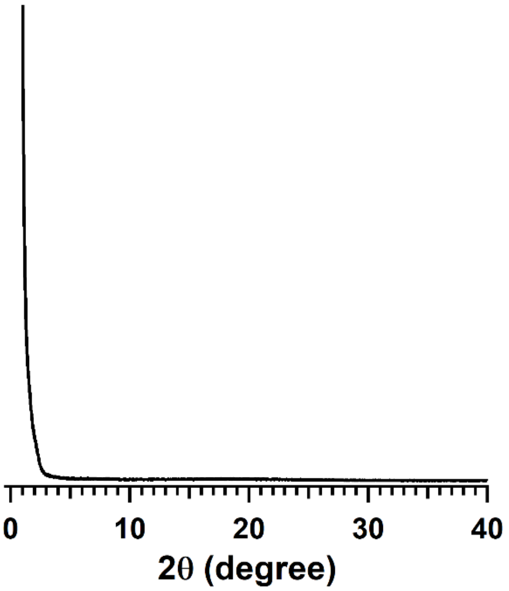

Supplementary Fig. 10 | PXRD patterns. PXRD of DBAAn-BTBA-HAPB-COF synthesized using one-pot method.

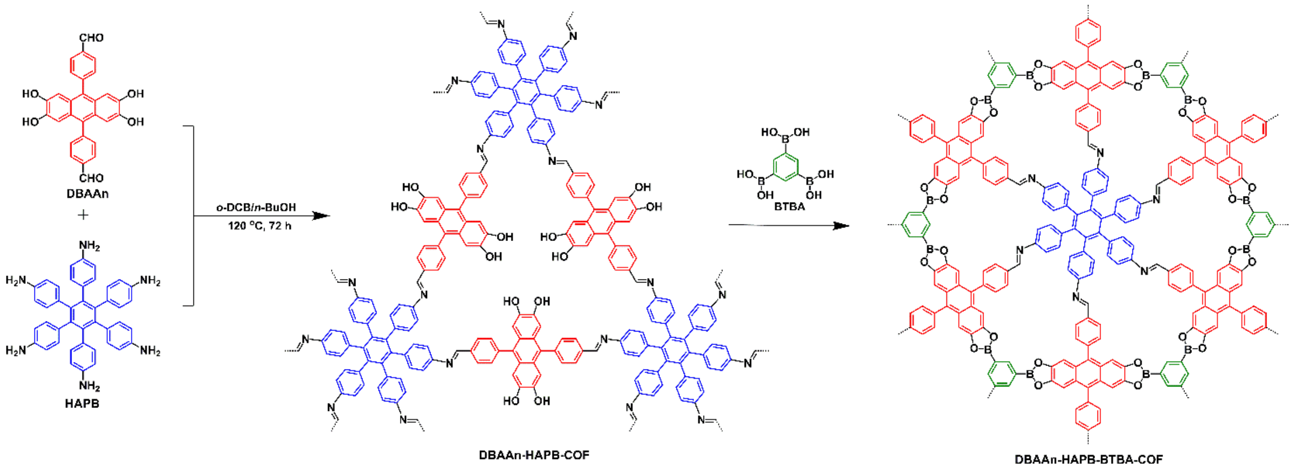

Supplementary Fig. 11 | Synthetic route. Schematic diagram for the synthesis of DBAAn-HAPB-COF and DBAAn-HAPB-BTBA-COF.

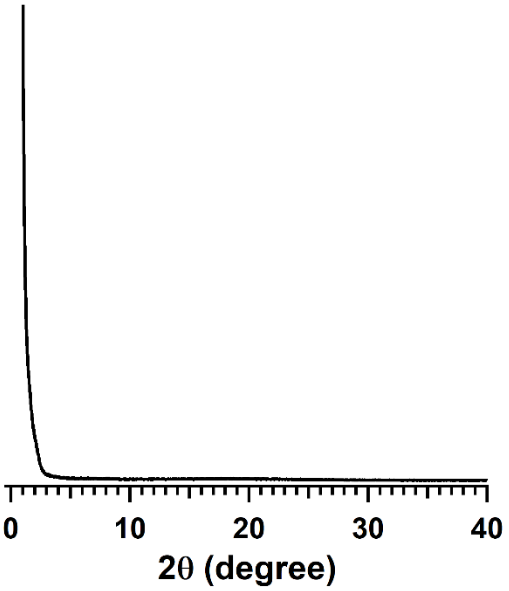

Supplementary Fig. 12 | PXRD pattern. PXRD patterns of amorphous DBAAn-HAPB-COF.

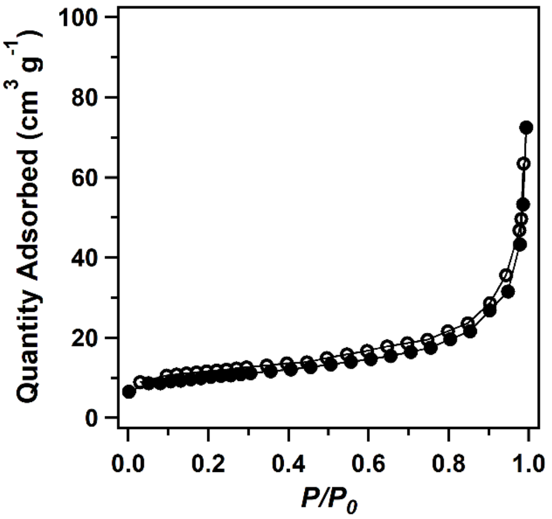

Supplementary Fig. 13 | Porosity. Nitrogen sorption isotherm of amorphous DBAAn-HAPB-COF.

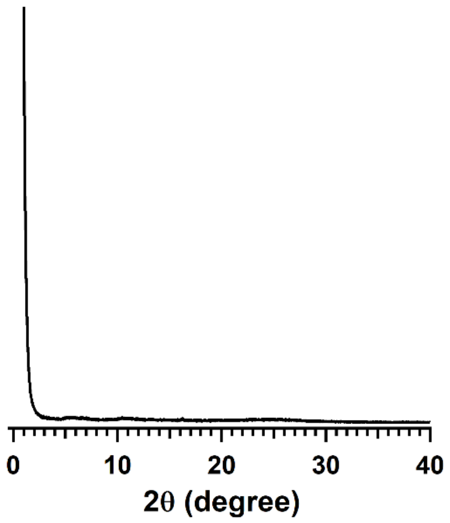

Supplementary Fig. 14 | PXRD pattern. PXRD patterns of amorphous DBAAn-BTBA-HAPB-COF.

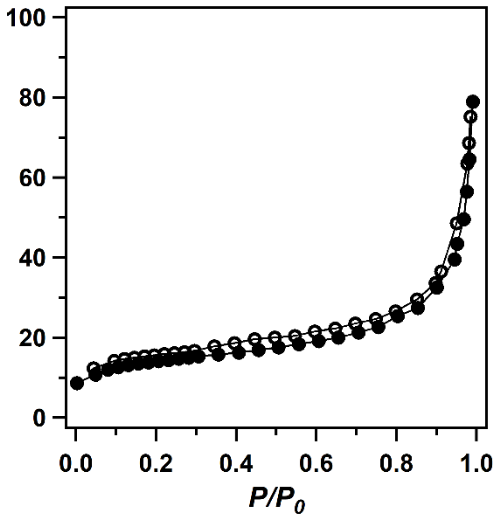

Supplementary Fig.15 | Porosity. Nitrogen sorption isotherm of amorphous DBAAn-HAPB-BTBA-COF.

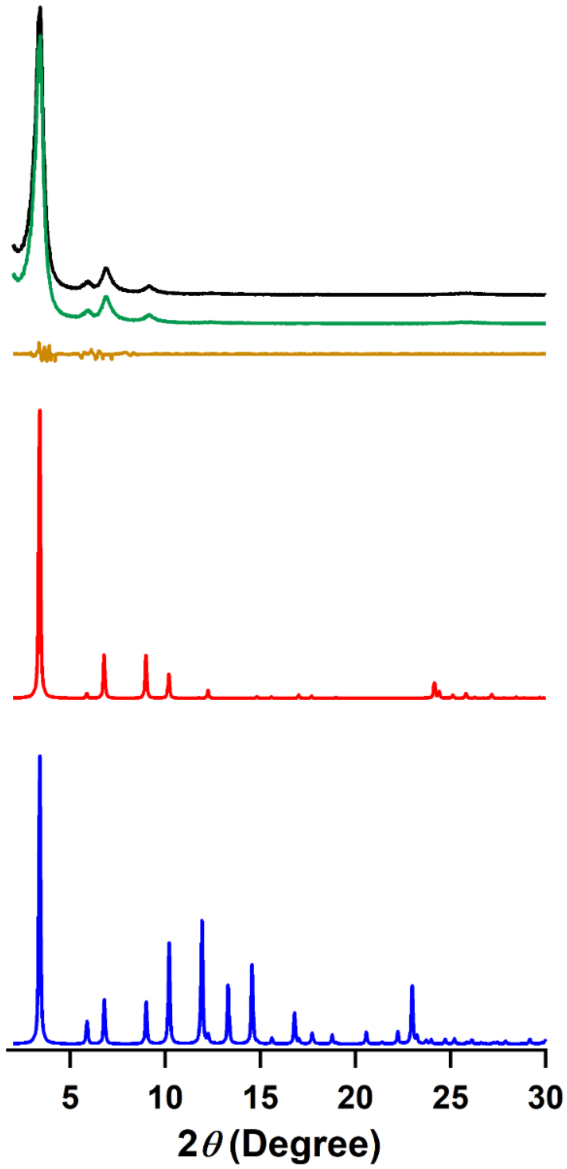

Supplementary Fig. 16 | PXRD patterns. PXRD patterns of DBAAn-BTBA-COF of experimentally observed curve (black curve), AA stacking (red curve), AB stacking (blue curve), Pawley refinement (green curve) and their difference (brown curve).

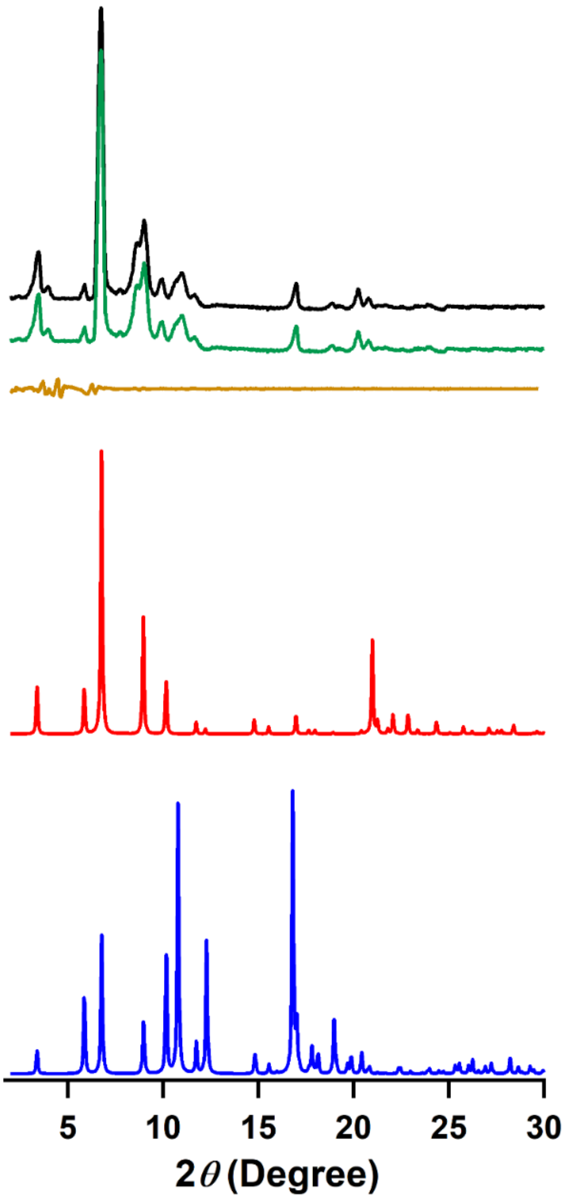

Supplementary Fig. 17 | PXRD patterns. PXRD pattern of DBAAn-BTBA-HAPB-COF of experimentally observed curve (black curve), AA stacking (red curve), AB stacking (blue curve), Pawley refinement (green curve) and their difference (brown curve).

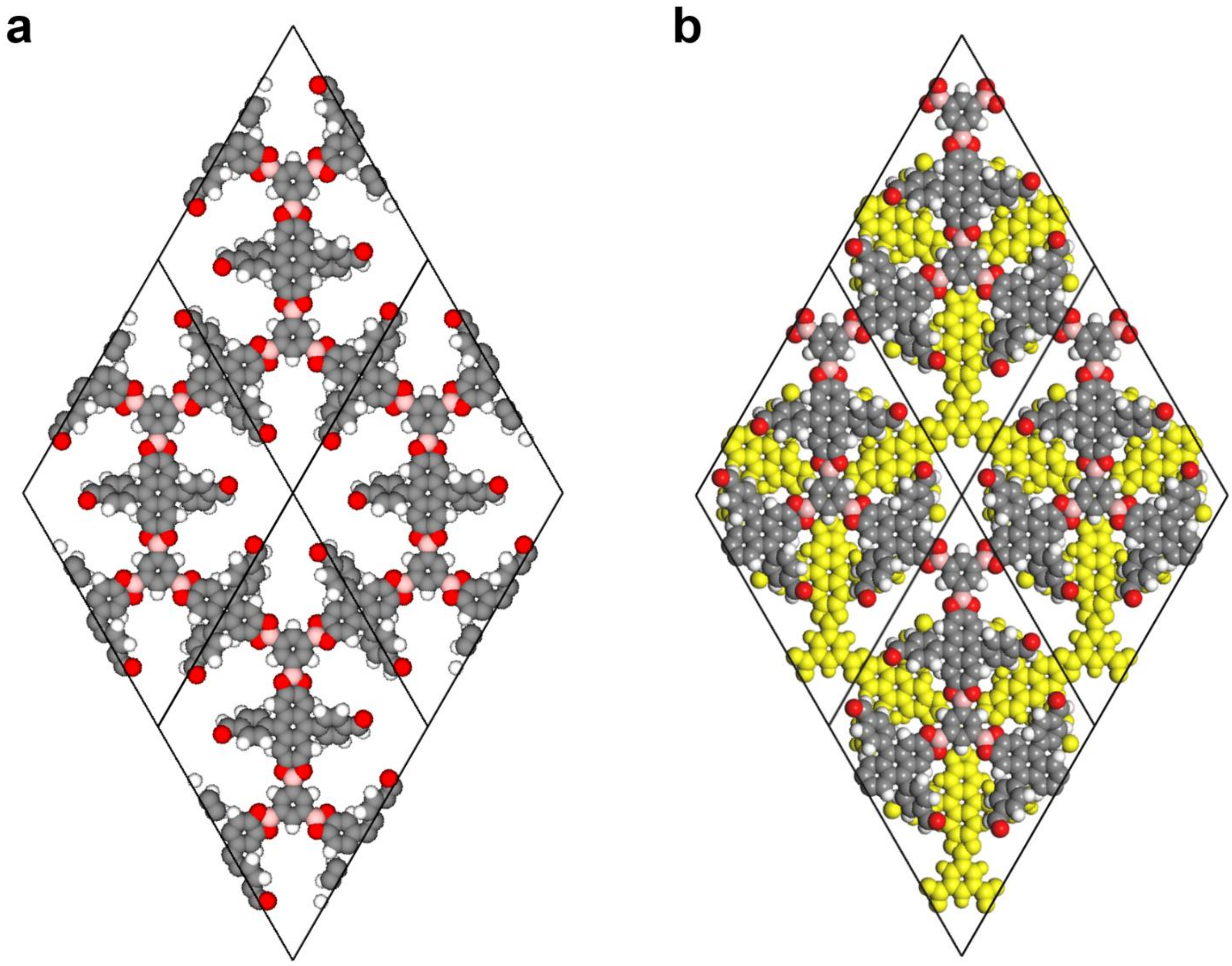

Supplementary Fig. 18 | Stacking mode. a, AA stacking mode of DBAAn-BTBA-COF. **b**, AB stacking mode of DBAAn-BTBA-COF.

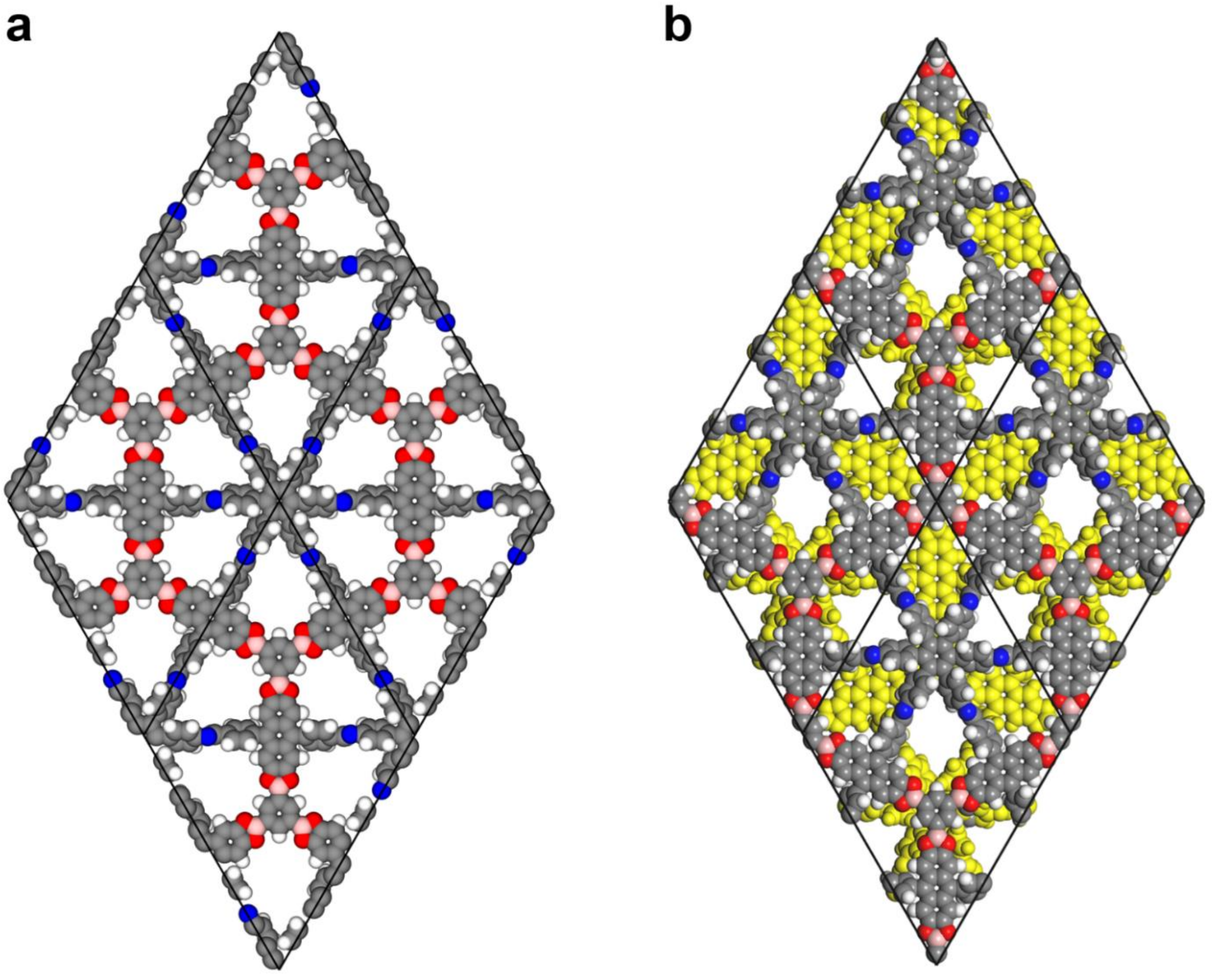

Supplementary Fig. 19 | Stacking mode. a, AA stacking mode of DBAAn-BTBA-HAPB-COF. **b**, AB stacking mode of DBAAn-BTBA-HAPB-COF.

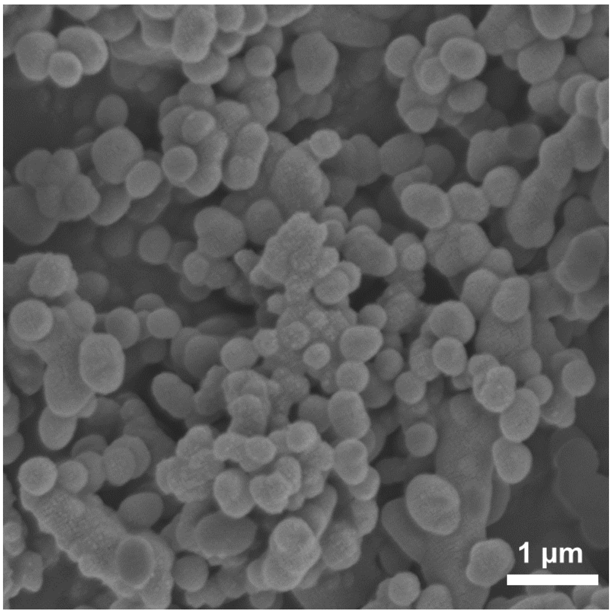

Supplementary Fig. 20 | SEM image. High-resolution SEM image of DBAAn-BTBA-COF.

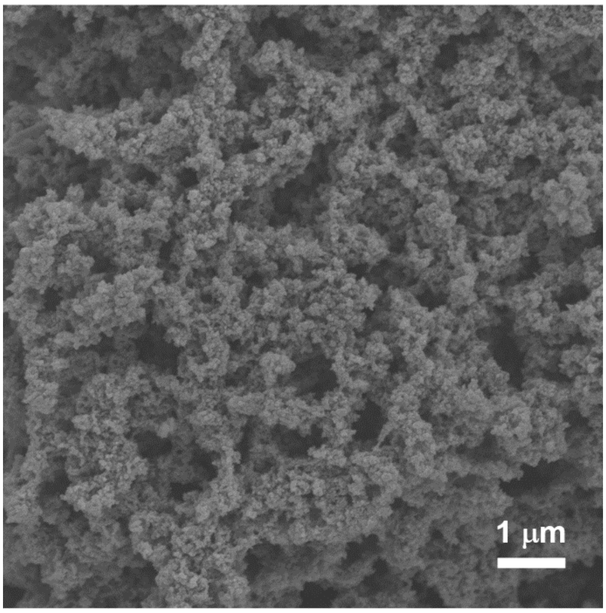

Supplementary Fig. 21 | SEM image. High-resolution SEM image of DBAAn-BTBA-HAPB-COF.

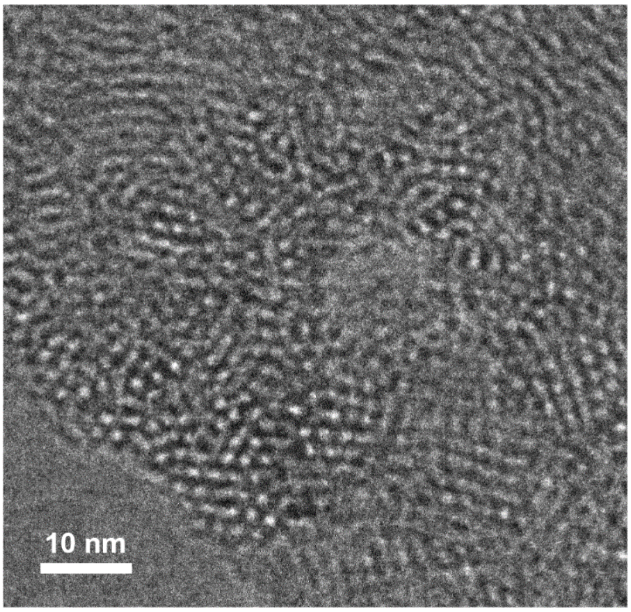

Supplementary Fig. 22 | TEM image. High-resolution TEM image of DBAAn-BTBA-COF.

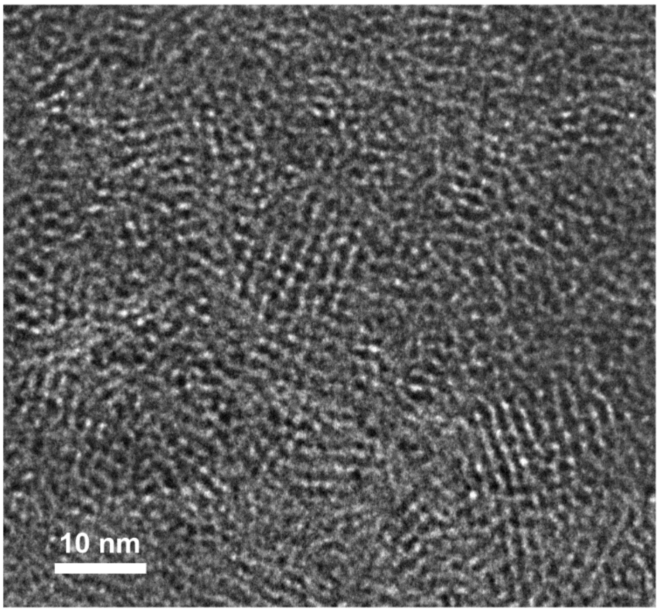

Supplementary Fig. 23 | TEM image. High-resolution TEM image of DBAAn-BTBA-HAPB-COF.

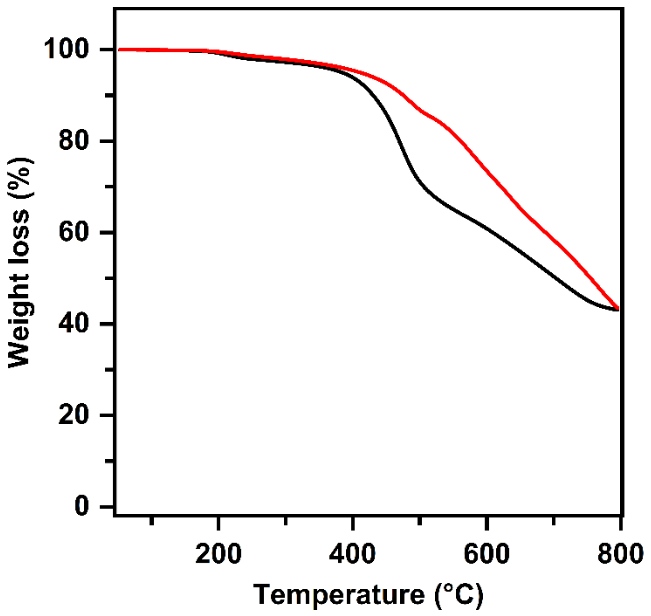

Supplementary Fig. 24 | TGA. TGA analysis curve of DBAAn-BTBA-COF (black curve) and DBAAn-BTBA-HAPB-COF (red curve).

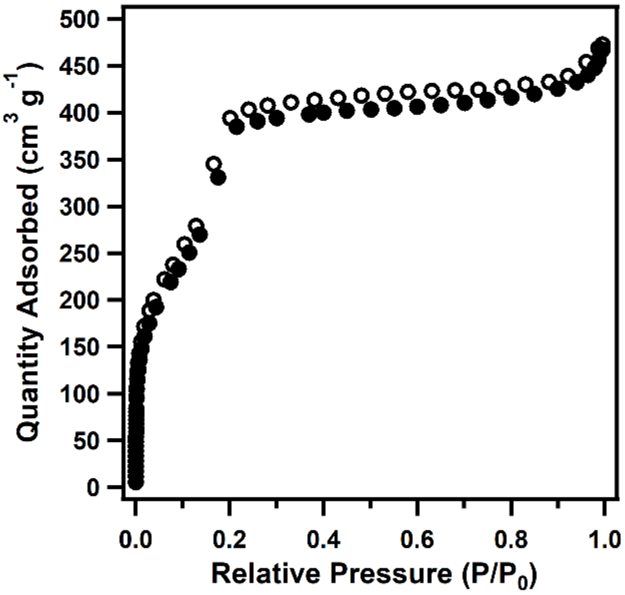

Supplementary Fig. 25 | Nitrogen sorption. Nitrogen sorption isotherm of DBAAn-BTBA -COF.

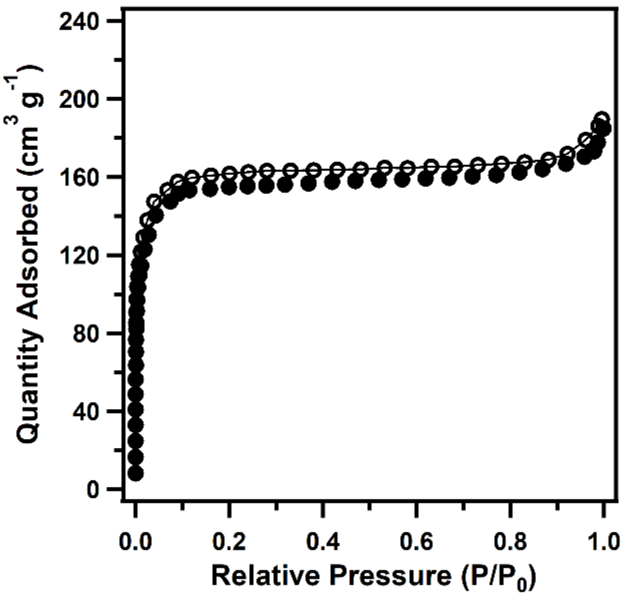

Supplementary Fig. 26 | Nitrogen sorption. Nitrogen sorption isotherm of DBAAn-BTBA-HAPB-COF.

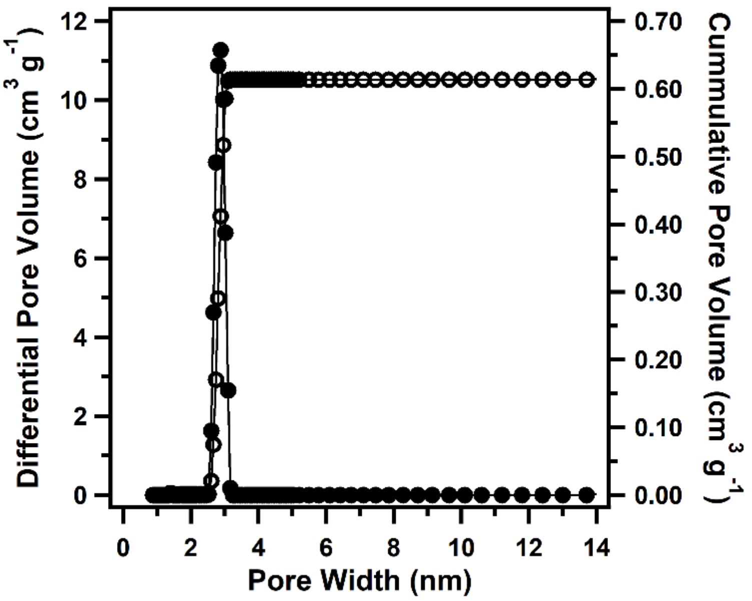

Supplementary Fig. 27 | Pore size. Pore size distribution profile of DBAAn-BTBA-COF.

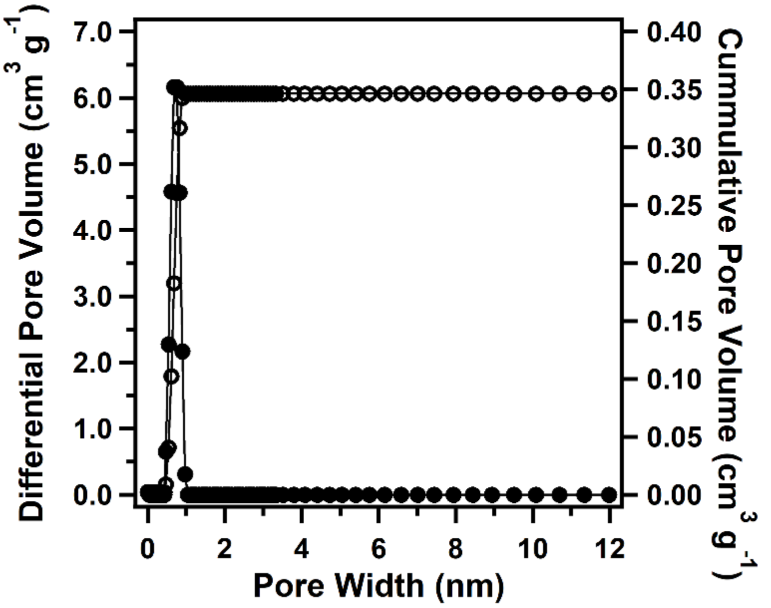

Supplementary Fig. 28 | Pore size. Pore size distribution profile of DBAAn-BTBA-HAPB-COF.

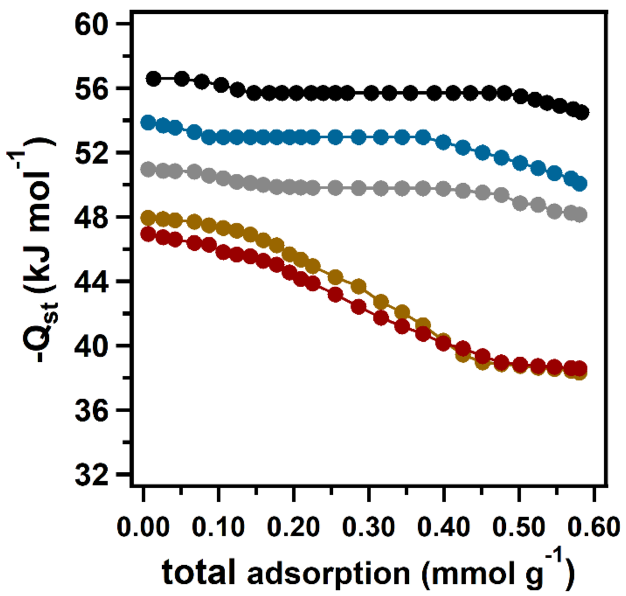

Supplementary Fig. 29 | Heat of adsorption. Experimental breakthrough data and calculated RON values of eluted hexane mixture at 1 bar and 120 °C (marron curve, 22DMB; brown curve, 23DMB; blue curve, 3MP; gray curve, 2MP; black curve, nHEX).

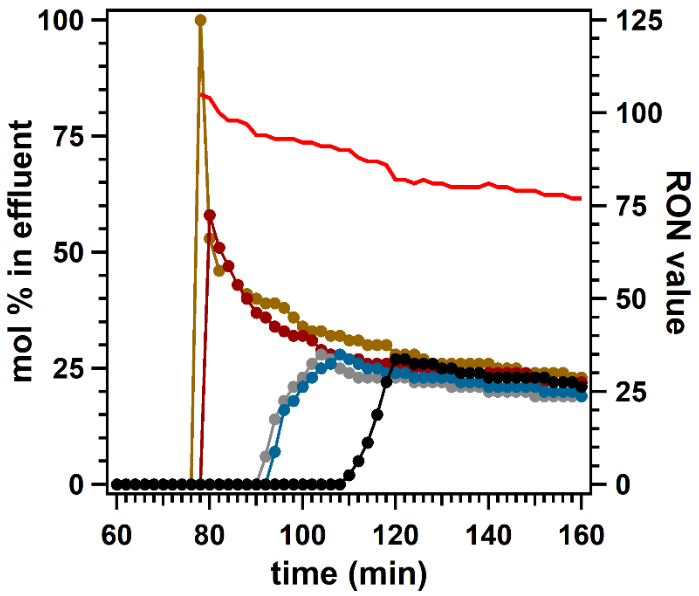

Supplementary Fig. 30 | Breakthrough curve. Breakthrough curves of an equimolar mixture of 22DMB (marron line), 23DMB (brown line), 2MP (blue line), 3MP (gray line), and nHEX (black line) in nitrogen at 1 atm and 100 °C, and these data sets correspond to the left Y axis. The red line is the calculated RON values for eluted mixture, which corresponds to the right Y axis.

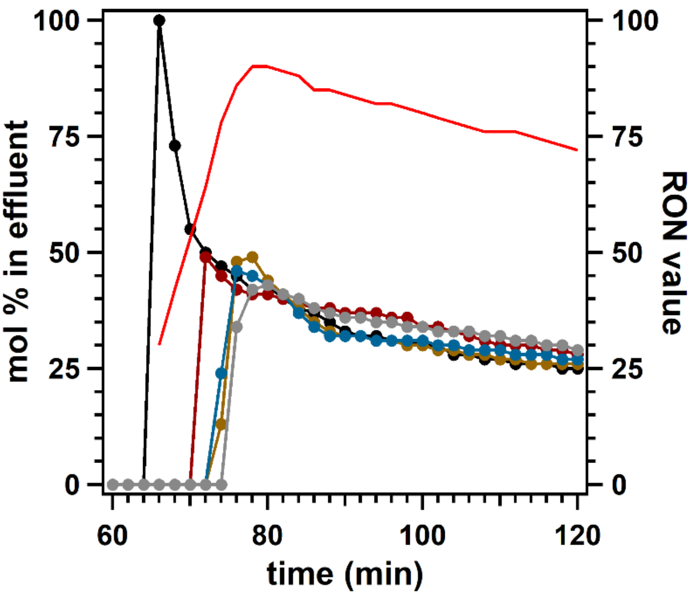

Supplementary Fig. 31 | Breakthrough curve. Breakthrough curves of an equimolar mixture of 22DMB (marron line), 23DMB (brown line), 2MP (blue line), 3MP (gray line), and nHEX (black line) in nitrogen at 1 atm and 150 °C, and these data sets correspond to the left Y axis. The red line is the calculated RON values for eluted mixture, which corresponds to the right Y axis.

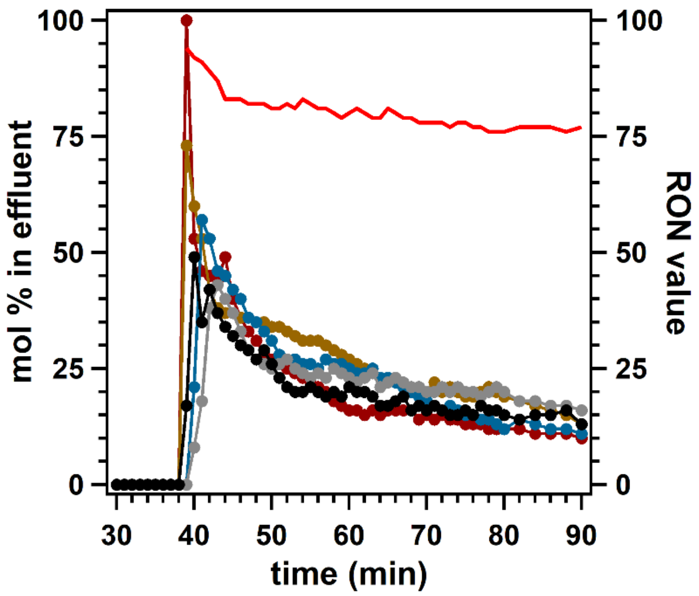

Supplementary Fig. 32 | Breakthrough curve. Breakthrough curves of an equimolar mixture of 22DMB (marron line), 23DMB (brown line), 2MP (blue line), 3MP (gray line), and nHEX (black line) for Ph-An-COF at 1 bar and 120 °C, and these data sets correspond to the left Y axis. The red line is the calculated RON values for eluted mixture, which corresponds to the right Y axis.

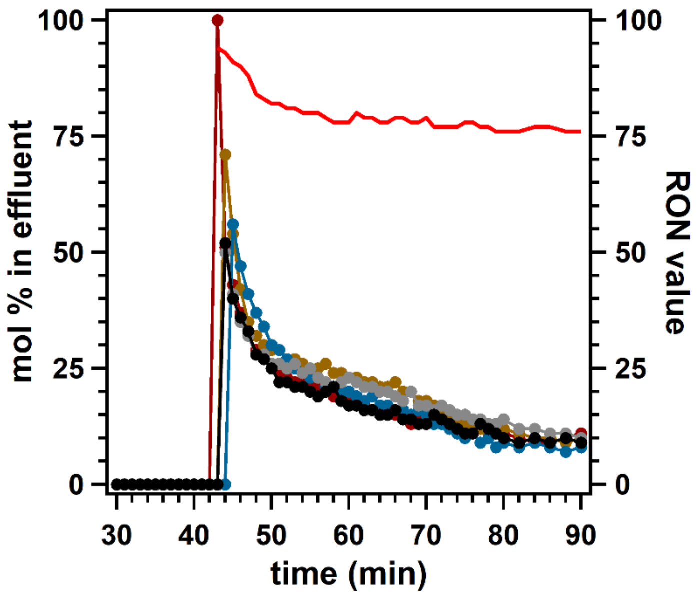

Supplementary Fig. 33 | Breakthrough curve. Breakthrough curves of an equimolar mixture of 22DMB (marron line), 23DMB (brown line), 2MP (blue line), 3MP (gray line), and nHEX (black line) for DBAAn-BTBA-COF at 1 bar and 120 °C, and these data sets correspond to the left Y axis. The red line is the calculated RON values for eluted mixture, which corresponds to the right Y axis.

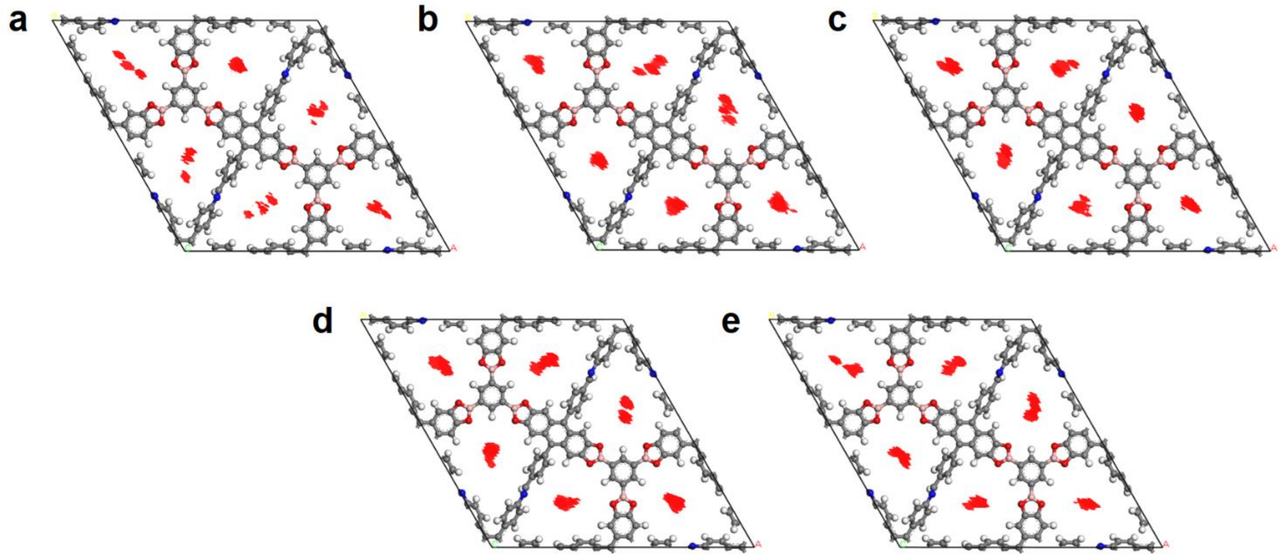

Supplementary Fig. 34 | Probability density diagrams. Calculated probability density diagrams for (a) 22DMB, (b) 23DMB, (c) 3MP, (d) 2MP, and (e) nHEX.

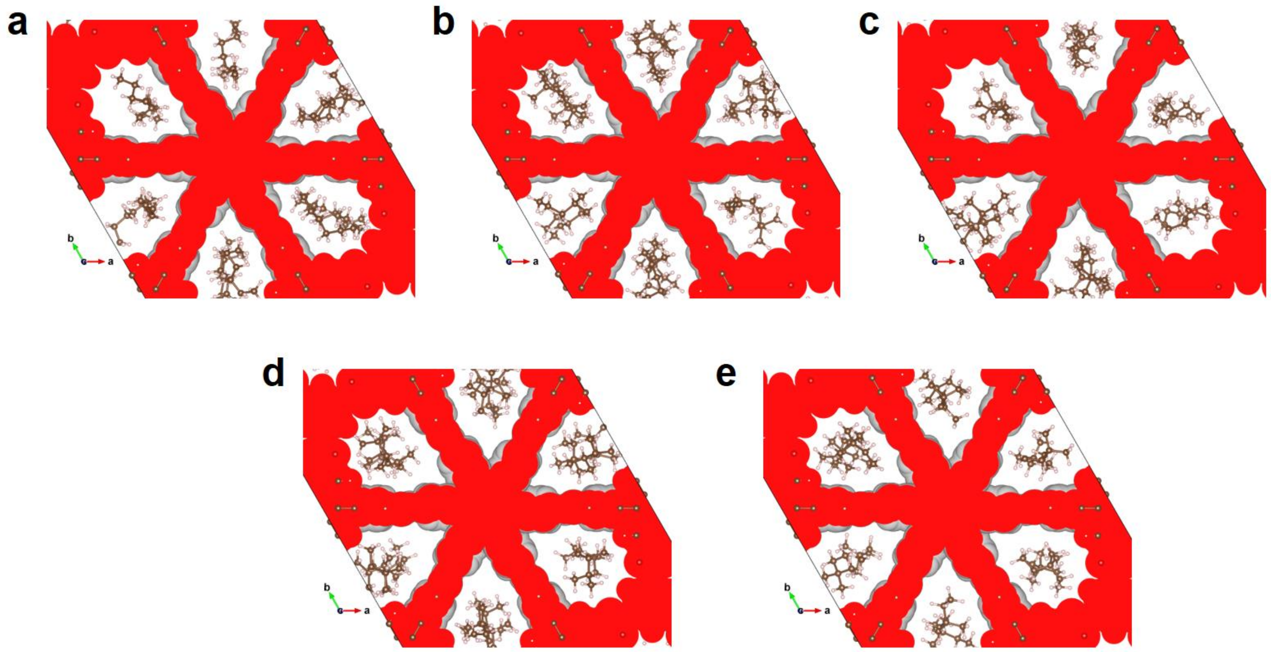

Supplementary Fig. 35 | Van der waals surfaces. Calculated van der waals surfaces of DBAAn-BTBA-HAPB-COF with (a) 22DMB, (b) 23DMB, (c) 3MP, (d) 2MP, and (e) nHEX inside the channels.

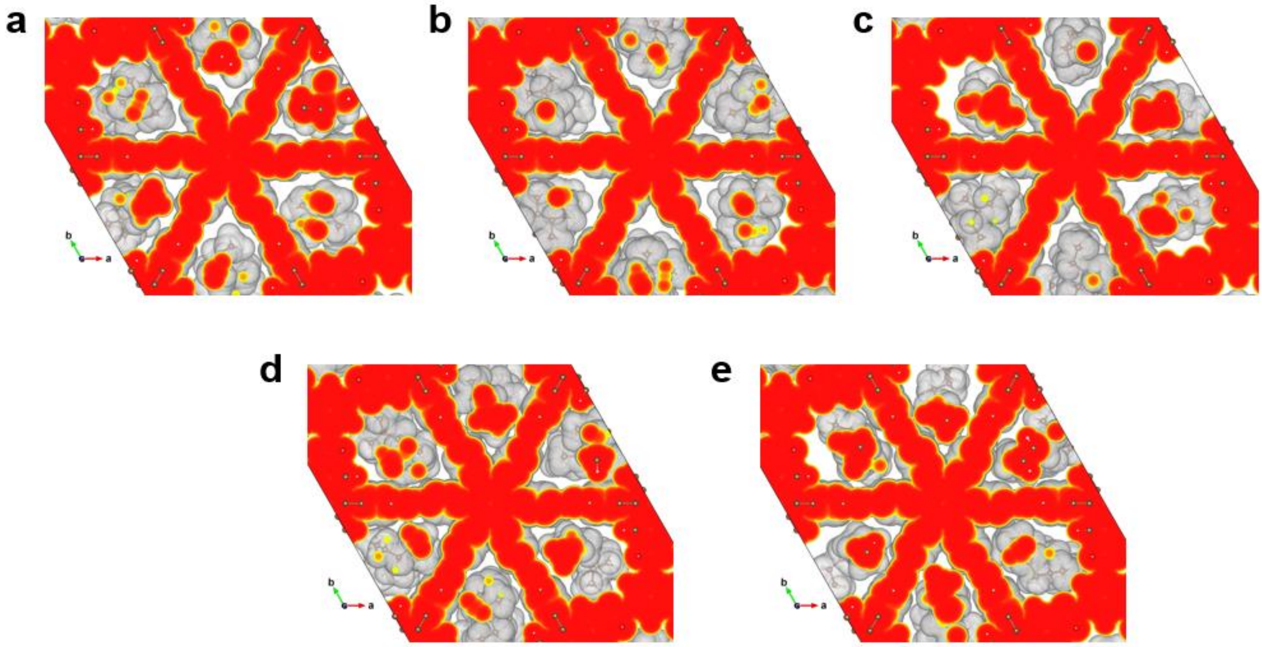

Supplementary Fig. 36 | Van der waals interaction. Calculated van der waals interactions between DBAAn-BTBA-HAPB-COF and (a) 22DMB, (b) 23DMB, (c) 3MP, (d) 2MP, and (e) nHEX.

Supplementary Tables

Supplementary Table 1 | Elemental analysis.

COFs		C (%)	H (%)	N (%)
DBAAn-BTBA-COF	Calculated	74.13	4.63	0.00
	Found	75.17	3.98	0.04
DBAAn-BTBA-HAPB-COF	Calculated	79.98	6.65	3.47
	Found	77.29	4.91	3.43

Supplementary Table 2 | Lattice parameters of DBAAn-BTBA-COF from Pawley refinement.

Formula	$C_{66}O_{12}B_6$
Formula weight	1048.86
Crystal system	hexagonal
Space group	*P*6 (No. 168)
Unit cell dimensions	a = 30.2358 Å, b = 30.2358 Å, c = 4.4387 Å,<br>α = β = 90°, and γ = 120°
Cell volume	2356.74 $Å^3$
Density calculated	0.64356 g cm^{-3}

Supplementary Table 3 | Atomic coordinates of DBAAn-BTBA-COF.

atom label	x	y	z	adp type	occupancy
H1	0.46773	0.37082	0.33621	Uiso	1
H2	0.37007	0.46561	0.64527	Uiso	1
C3	0.27884	0.64023	0.48768	Uiso	1
C4	0.30572	0.61299	0.48767	Uiso	1
B5	0.38889	0.60856	0.48712	Uiso	1
O6	0.36728	0.55679	0.57515	Uiso	1
C7	0.40672	0.54597	0.53805	Uiso	1
C8	0.45181	0.59132	0.43654	Uiso	1
O9	0.44064	0.63043	0.39861	Uiso	1
C10	0.40555	0.50026	0.57625	Uiso	1
C11	0.45204	0.49891	0.51321	Uiso	1
C12	0.50003	0.54728	0.46605	Uiso	1
C13	0.49788	0.59326	0.40134	Uiso	1
C14	0.45209	0.45159	0.49266	Uiso	1
C15	0.40316	0.40158	0.50468	Uiso	1
C16	0.35888	0.39318	0.32265	Uiso	1
C17	0.31314	0.34622	0.34227	Uiso	1
C18	0.31048	0.30566	0.53811	Uiso	1
C19	0.35445	0.31302	0.71556	Uiso	1
C20	0.39947	0.36023	0.70256	Uiso	1
C21	0.74378	0.00516	0.56903	Uiso	1
O22	0.77753	0.03234	0.77277	Uiso	1
H23	0.77023	0.74803	0.39537	Uiso	1
H24	0.28405	0.57099	0.48746	Uiso	1
H25	0.27934	0.34028	0.19781	Uiso	1
H26	0.3516	0.28151	0.87245	Uiso	1
H27	0.5765	0.93759	0.15648	Uiso	1
H28	0.63366	0.06625	0.85392	Uiso	1

Supplementary Table 4 | Lattice parameters of DBAAn-BTBA-HAPB-COF from Pawley refinement.

Formula	$C_{82}O_{12}B_6N_6$
Formula weight	1324.57
Crystal system	hexagonal
Space group	*P*6 (No. 168)
Unit cell dimensions	a = 30.1380 Å, b = 30.1380 Å, c = 4.2305 Å,<br>α = β = 90°, and γ = 120°
Cell volume	2046.56 Å^3
Density calculated	0.68483 g cm^{-3}

Supplementary Table 5 | Atomic coordinates of DBAAn-BTBA-HAPB-COF.

atom label	x	y	z	adp type	occupancy
C1	0.50421	0.90788	0.63612	Uiso	1
C2	0.50327	0.95468	0.60866	Uiso	1
C3	0.45435	0.95191	0.59109	Uiso	1
C4	0.40959	0.9024	0.56298	Uiso	1
C5	0.54878	0.00286	0.60018	Uiso	1
C6	0.59967	0.00602	0.60052	Uiso	1
C7	0.63891	0.03922	0.79663	Uiso	1
C8	0.68636	0.04235	0.79663	Uiso	1
C9	0.69711	0.01243	0.60018	Uiso	1
C10	0.6579	0.97904	0.4048	Uiso	1
C11	0.61051	0.97601	0.40472	Uiso	1
C12	0.74745	0.01581	0.6026	Uiso	1
N13	0.76378	0.00016	0.3812	Uiso	1
C14	0.99938	0.95288	0.41492	Uiso	1
C15	0.90284	0.90411	0.41191	Uiso	1
C16	0.86375	0.89542	0.20785	Uiso	1
C17	0.81744	0.84984	0.20312	Uiso	1
C18	0.80748	0.80982	0.40369	Uiso	1
C19	0.84668	0.818	0.6067	Uiso	1
C20	0.89264	0.864	0.6113	Uiso	1
H21	0.63189	0.06237	0.9618	Uiso	1
H22	0.71588	0.06875	0.95585	Uiso	1
H23	0.66476	0.95489	0.24678	Uiso	1
H24	0.58155	0.95039	0.24038	Uiso	1
H25	0.77149	0.03413	0.80688	Uiso	1
H26	0.8698	0.92491	0.03622	Uiso	1
H27	0.78789	0.84537	0.04024	Uiso	1
H28	0.84098	0.78755	0.76613	Uiso	1
H29	0.92137	0.86892	0.78564	Uiso	1
C30	0.40077	0.53967	0.62208	Uiso	1
C31	0.44611	0.58771	0.57629	Uiso	1
O32	0.35265	0.53758	0.64994	Uiso	1
O33	0.4409	0.63227	0.54906	Uiso	1
H34	0.3682	0.45893	0.6691	Uiso	1
H35	0.52752	0.62794	0.52985	Uiso	1
H36	0.47551	0.66011	0.52019	Uiso	1
H37	0.36091	0.57286	0.62979	Uiso	1

Supplementary Table 6 | Adsorption and separation of hexane isomers on selected porous materials.

Porous materials	Isomers tested (uptake, mmol·g^{-1})	T (°C)	Separation mechanism	Experimental method	Ref.
DBAAn-BTBA-HAPB-COF	*n*HEX (0.96), 2MP (0.80), 3MP (0.76), 23DMB (0.73), 22DMB (0.72)	120	molecular sieving	adsorption isotherm/ breakthrough	This work
$Fe_2(bdp)_3$	nHEX (1.32), 2MP (1.18), 3MP (1.27), 23DMB (1.37), 22DMB (1.22)	160	thermodynamic	adsorption isotherm/ breakthrough	S1
MIL-101(Cr)	nHEX (10.2), 2MP (9.2), 22DMB (8.6), 23DMB (8.4)	40	thermodynamic	adsorption isotherm/gas chromatography	S2
Zr-bptc	nHEX (1.51), 3MP (0.29), 23DMB (0)	150	molecular sieving	adsorption isotherm/ breakthrough	S3
MIL-53(Fe)-$(CF_3)_2$	nHEX (0.37), 3MP (0.35), 22DMB (0.31)	40	kinetic	adsorption isotherm/ breakthrough	S4
Y-fum-fcu MOF	nPEN (1.87), 2MB (0)	20	molecular sieving	adsorption isotherm/ breakthrough	S5
ZIF-8	nHEX (2.63), 2MP (2.47), 23DMB (1.88), 22DMB (0.20)	100	kinetic	adsorption isotherm	S6
Zr-abtc	nHEX (1.28), 3MP (1.02), 23DMB (0.58)	150	thermodynamic	adsorption isotherm/ breakthrough	S3
$Zn_2(Hbdc)_2(dmtrz)_2$	nHEX (1.53), 3MP (1.40), 22DMB (0.40)	25	kinetic	adsorption isotherm/gas chromatography	S7
$Ca(H_2tcpb)$	nHEX (1.62), 3MP (1.54), 22DMB (0.10)	60	molecular sieving	adsorption isotherm/ breakthrough	S8
ZIF-69	nHEX (3.95), 2MP (1.16)	25	kinetic	adsorption isotherm/ breakthrough	S9
$Fe_3(\mu_3\text{-O})](6fdca)_3$	nHEX (0.75), 3MP (0.65), 22DMB (0.05)	25	kinetic	adsorption isotherm/ breakthrough	S10

Supplementary References

S1. Herm, Z. R. *et al*. Separation of hexane isomers in a metal-organic framework with triangular channels. *Science* **340**, 960−964 (2013).

S2. Belarbi, H. *et al*. Adsorption and separation of hydrocarbons by the metal organic framework MIL-101(Cr). *Colloids Surf. A* **520**, 46−52 (2017).

S3. Wang, H. *et al*. Topologically guided tuning of Zr-MOF pore structures for highly selective separation of C6 alkane isomers. *Nat. Commun.* **9**, 1745 (2018).

S4. Mendes, P. A. P. *et al*. Complete separation of hexane isomers by a functionalized flexible metal organic framework. *Adv. Funct. Mater.* **24**, 7666−7673 (2014).

S5. Assen, A. H. *et al*. Ultra-tuning of the rare-earth fcu-MOF aperture size for selective molecular exclusion of branched paraffins. *Angew. Chem. Int. Ed.* **54**, 14353−14358 (2015).

S6. Ferreira, A. F. P. *et al*. Sieving dibranched from mono-branched and linear alkanes using ZIF-8: experimental proof and theoretical explanation. *Phys. Chem. Chem. Phys.* **15**, 8795−8804 (2013).

S7. Ling, Y. *et al*. A zinc(ii) metal-organic framework based on triazole and dicarboxylate ligands for selective adsorption of hexane isomers. *Chem. Commun.* **47**, 7197−7199 (2011).

S8. Wang, H. *et al*. One-of-a-kind: a microporous metal−organic framework capable of adsorptive separation of linear, mono- and di-branched alkane isomers via temperature- and adsorbate-dependent molecular sieving. *Energy Environ. Sci.* **11**, 1226−1231 (2018).

S9. Chen, L. *et al*. Effective Adsorption Separation of n-Hexane/2-Methylpentane in Facilely Synthesized Zeolitic Imidazolate Frameworks ZIF-8 and ZIF-69. *Ind. Eng. Chem. Res.* **55**, 10751−10757 (2016).

S10. Lv, D. *et al*. Iron-Based Metal−Organic Framework with Hydrophobic Quadrilateral Channels for Highly Selective Separation of Hexane Isomers. *ACS Appl. Mater. Interfaces* **10**, 6031−6038 (2018).
